# Supplementary material for: The ribosome-associated N-terminal acetyltransferase B coordinates global proteostasis and autophagy in plants by creating Ac/N-degrons
Source: Nat Commun. 2026 Mar 31;17:3116. doi: 10.1038/s41467-026-71208-2 (PMC13039445; doi:10.1038/s41467-026-71208-2)
Supplement: Supplementary file 1 — Supplementary Information [file 41467_2026_71208_MOESM1_ESM.pdf]

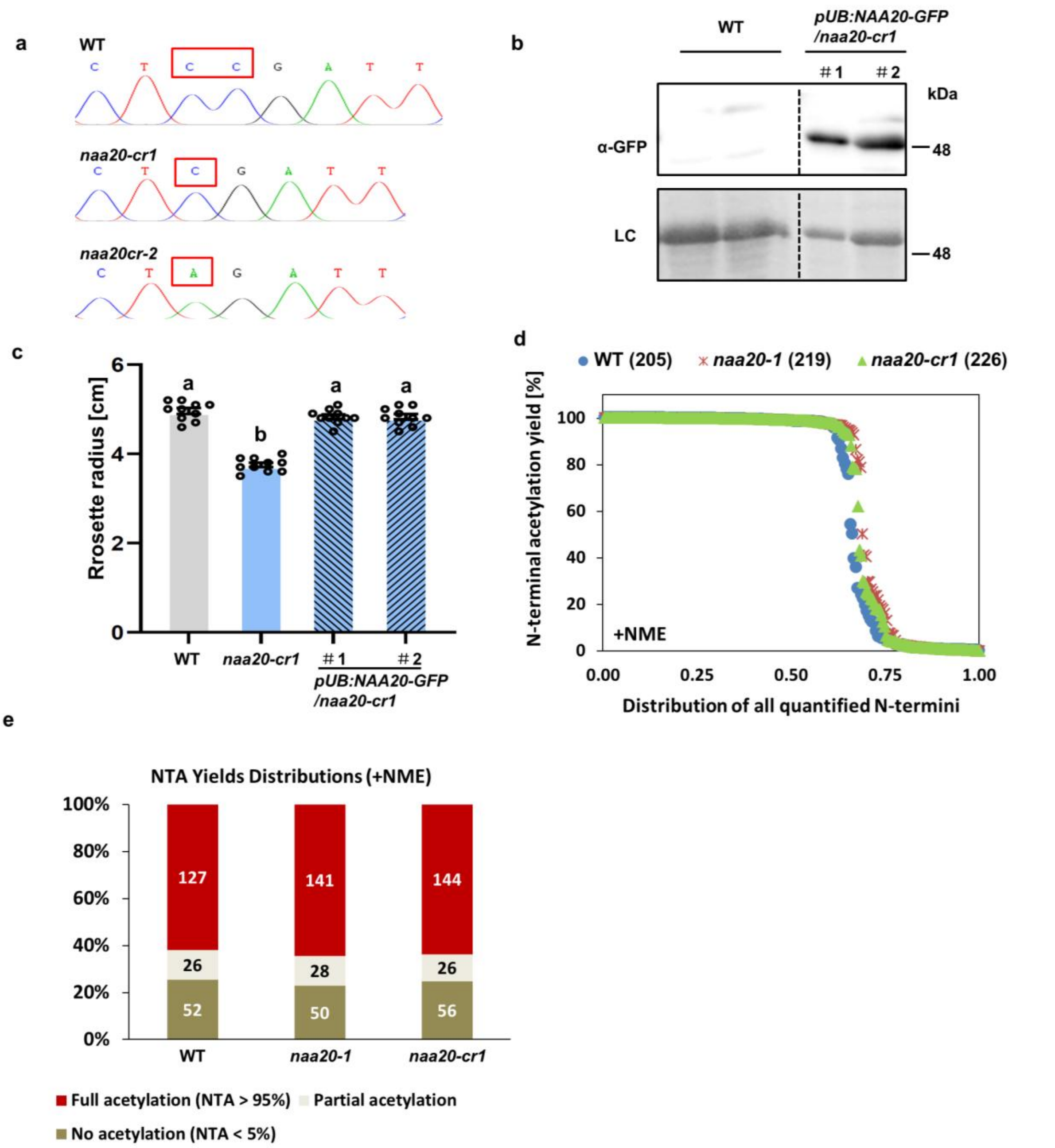

Supplementary Fig. 1 Knockout of NAA20 leads to retarded plant development and decreased N-acetylome

a Sequencing of the *NAA20* gene in the wild type (WT) and two *NAA20* CRISPR/Cas9 mutants. **b** Immunoblot detection of NAA20-GFP fusion protein in two independent *pUB:NAA20-GFP/naa20-cr1* transgenic lines. Wild type (WT) was used as a control to assess the specificity of the GFP antibody. Amido black staining of proteins transferred to the PVDF membrane served as a loading control (LC). **c** Rosette radius of 6-week-old soil-grown wild type (WT), *naa20-cr1* and two independent *pUB:NAA20-GFP/naa20-cr1* transgenic lines expressing the full-length *NAA20* CDS fused with GFP. Data are shown as means ± SEM. Circles indicate individual data points. Different letters indicate individual groups identified by pairwise multiple comparisons with a one-way ANOVA followed by a Tukey's test ($p < 0.05$, $n = 10$ individual plants) **d** Distribution for NTA yields of peptides that were subject to iMet excision (+NME) in leaves of wild type (WT) and *naa20* mutants as determined by the

SILProNAQ. Numbers in brackets display the quantity of detected N termini. **e** Comparison of fully, partially, and non-acetylated protein N termini that were subject to iMet excision (+NME) in leaves of wild type (WT) and the *naa20* mutants. Numbers in the bars represent the quantity of characterized N termini in this fraction. Source data are provided as a Source Data file.

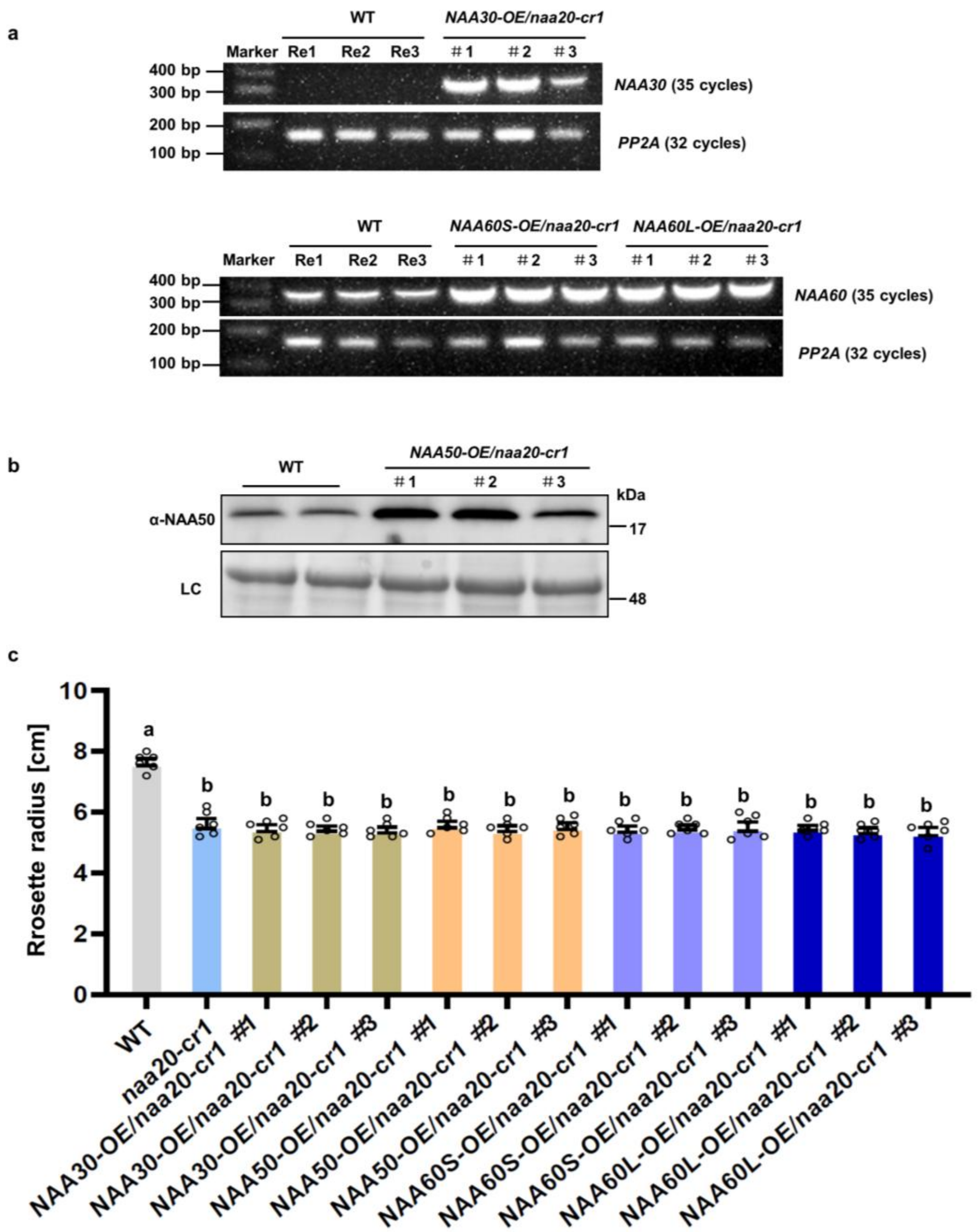

Supplementary Fig. 2 Overexpression of NatC, NatE and NatF cannot rescue the developmental defects of *naa20-cr1*

a RT-PCR analysis of the transgenic lines showing the overexpression of *NAA30*, *NAA60S* and *NAA60L* in *naa20-cr1* backgrounds, respectively. *PP2A* (*AT1G69960*) served as the reference genes. **b** Immunoblot detection of NAA50 in three independent *pUB:NAA50/naa20-cr1* transgenic lines. Amido black staining of proteins transferred to the PVDF membrane served as loading control (LC). **c** Rosette radius of 8-week-old soil-grown wild type (WT), *naa20-cr1*, and transgenic lines overexpressing *NAA30*, *NAA50*, *NAA60S* and *NAA60L* in *naa20-cr1* backgrounds, respectively. Data are shown as means ± SEM. Circles indicate individual data points. Different letters indicate individual groups identified by pairwise multiple comparisons with a one-way ANOVA followed by a Tukey's test ($p < 0.05$, $n = 6$ biologically independent samples). Scale bar, 2 cm. Source data are provided as a Source Data file.

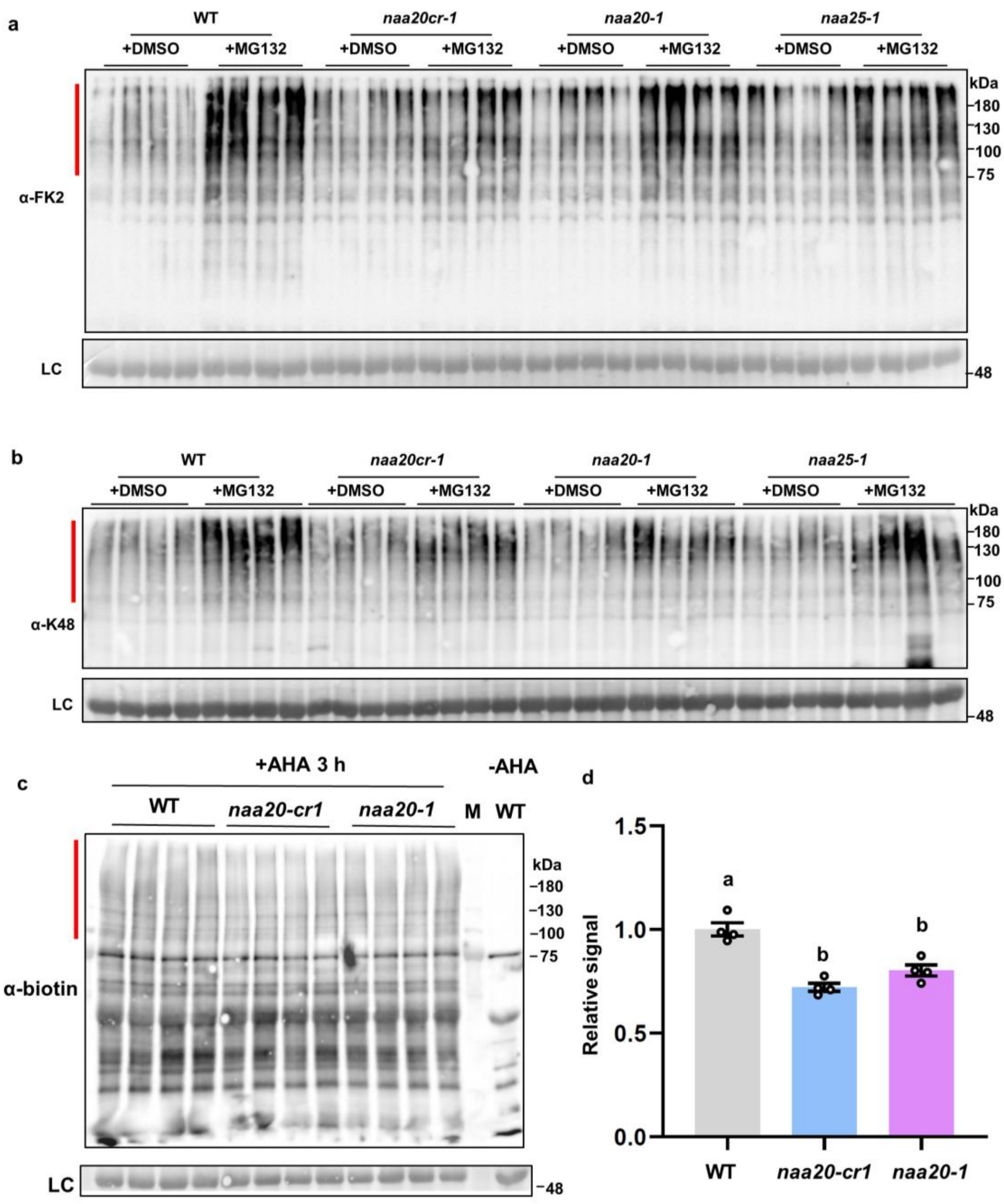

Supplementary Fig. 3 NatB positively regulates protein turnover in plants

a, b Immunoblot detection of global mono- and poly-ubiquitination levels with the specific FK2-ubiquitin antibody (**a**) and global K48-linked poly-ubiquitination levels with the specific K48-ubiquitin antibody (**b**) in the wild type (WT) and *natb* mutants in the presence or absence of the proteasome inhibitor MG132. **c, d** Comparison of azidohomoalanine incorporation into foliar proteins of wild type (WT) and *naa20* mutants. Newly translated proteins were labeled with 50 µM azidohomoalanine by floating leaf discs of six-week-old wild type (WT) and *naa20* mutants for 3 h on ½ Hoagland medium. Azidohomoalanine incorporation into proteins derived from wild type (WT) and *naa20* leaves was detected (**c**) and quantified (**d**) after azidohomoalanine-mediated biotin labeling and immunoblotting with the specific Neutravidin-HRP. DMSO treated wild-type leaf discs were used as a control to show endogenous biotinylated proteins. The red bar indicates the area of the western blot used for quantification. Data are shown as means ± SEM (n = 4 biologically independent samples). Circles indicate individual data points. Different letters indicate individual groups

identified by pairwise multiple comparisons with a one-way ANOVA followed by a Tukey´s test ($P < 0.05$). In (**a**-**c**), amido black staining of proteins transferred to the PVDF membrane served as loading control (LC) and all experiments were repeated three times with consistent results. Source data are provided as a Source Data file.

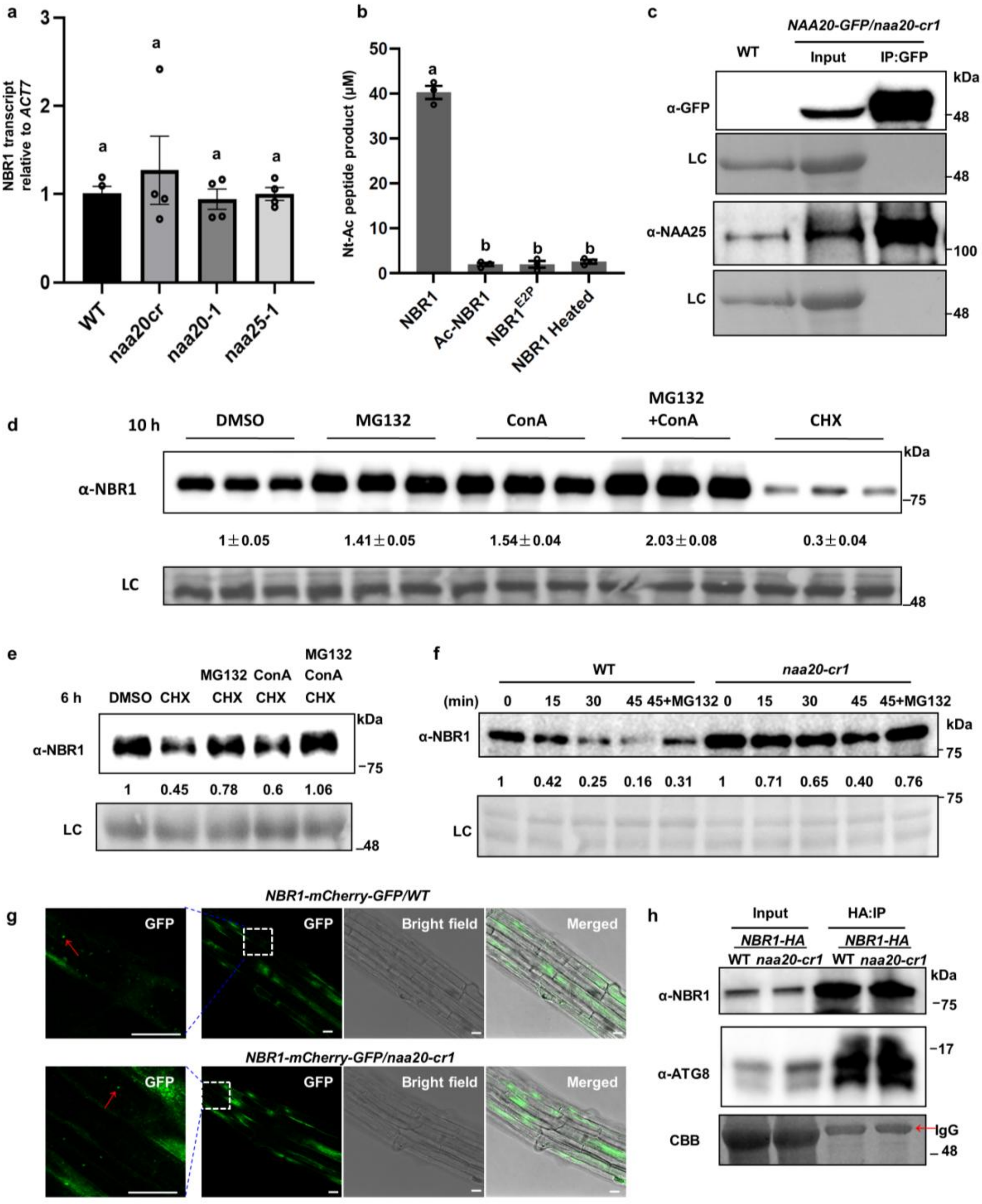

Supplementary Fig. 4 NBR1 is a substrate of NatB and may undergo both autophagic degradation and proteasomal degradation

a The transcript levels of NBR1 in the wild type (WT) and *natb* mutants determined by qRT-PCR. *Actin7* (*At5g09810*) served as the reference genes. *n* = 4 biologically independent samples. **b** DTNB-based *in vitro* Nt-acetylation assay confirms NBR1 as NatB substrates. The NBR1^{E2P} variant, N-terminally acetylated NBR1 (Ac-NBR1) peptides, and NBR1 peptides incubated with heated NatB were used as negative controls. *n* = 3 biologically independent samples. **c** Immunoblot detection of the NatB complex (NAA20 and NAA25) used in the DTNB-based *in vitro* Nt-acetylation assay. Immunopurified proteins using GFP beads from N*AA20-GFP/naa20-cr*1 transgenic plants were subjected to immunoblot detection with GFP and NAA25 antibodies. **d** Immunoblot detection of NBR1 in wild type after 10 h treatment

with different drugs as indicated. *n* = 3 biologically independent samples. **e** Immunoblot detection of NBR1 in *naa20-cr1* seedling after 6 h treatment with different drugs as indicated. **f** Immunoblot detection of NBR1 degradation rate in the wild type (WT) and *naa20-cr1* by a cell-free degradation assay. 50 µM of MG132 was applied to inhibit UPS-mediated protein degradation. The protein levels were normalized to the value at 0 min (set to 1). **g** Subcellular localization of NBR1 in root cells of 7-day-old *NBR1-mCherry-GFP/WT* and *NBR1mCherry-GFP/naa20-cr1* seedlings. The red arrows indicate the punctate localization pattern of NBR1. Scale bar, 20 µm. **h** Detection of the interaction between NBR1 and ATG8 in *NBR1-HA/WT* and *NBR1-HA/naa20-cr1* transgenic plants. Total proteins stained with Coomassie Brilliant Blue (CBB) were used as the internal control for input. The red arrow indicates IgG attached to HA magnetic beads. In (**a**, **b**), data are shown as means ± SEM, and different letters indicate individual groups identified by pairwise multiple comparisons with a one-way ANOVA followed by a Tukey´s test ($P < 0.05$). In (**c**-**f**), amido black staining of proteins transferred to the PVDF membrane served as a loading control (LC), and all experiments were repeated three times with consistent results. In (**d**, **e**), the protein levels were normalized to the value of DMSO treated sample (set to 1). Source data are provided as a Source Data file.

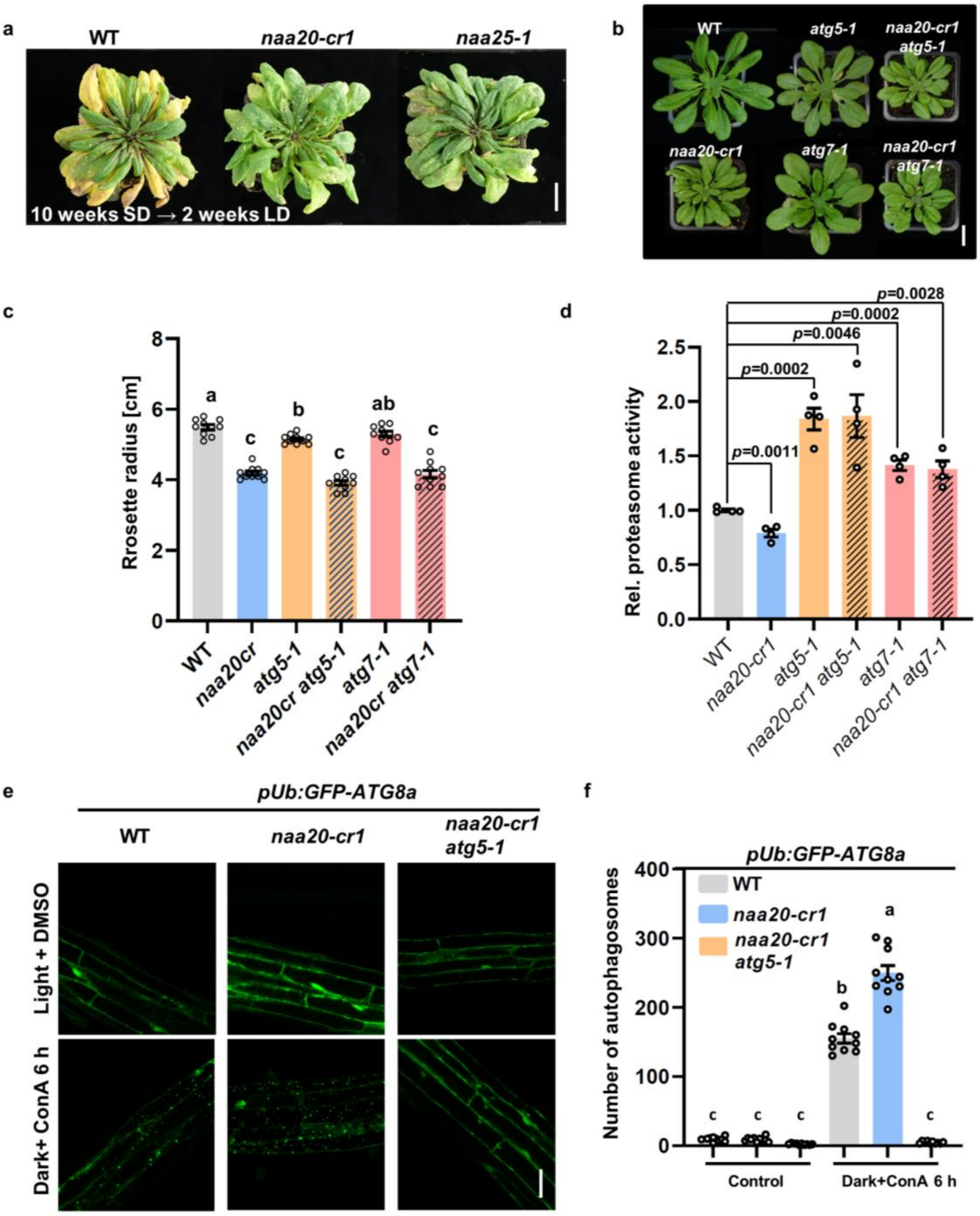

Supplementary Fig. 5 Autophagy induction in *naa20* is critical for dark resistance phenotypes

a Representative phenotype of 12-week-old soil-grown wild type (WT), *naa20-cr1*, and *naa25-1* plants grown for ten weeks in short-day conditions, followed by two weeks of long-day conditions. Scale bar, 2 cm. **b**, **c** Representative phenotype (**b**) and rosette radius (**c**) of 7-week-old soil-grown wild type (WT), *naa20-cr1*, *atg5-1*, *atg7-1*, *naa20-cr1 atg5-1*, and *naa20-cr1 atg7-1* plants. *n* = 10 plants. Scale bar, 2 cm. **d** Quantification of proteasome activity in leaves of 7-week-old soil-grown wild type (WT), *naa20-cr1*, *atg5-1*, *atg7-1*, *naa20-cr1 atg5-1*, and *naa20-cr1 atg7-1* plants. Data are shown as means ± SEM (*n* = 4 biologically independent samples). Circles indicate individual data points. *P*-values were calculated by a two-tailed Student's t-test. **e** Confocal microscopy of autophagic bodies in root cells. Seven-day-old *pUBQ:GFP-ATG8a/WT*, *pUBQ:GFP-ATG8a/naa20-cr1,* and *pUBQ:GFP-ATG8a/naa20-cr1 atg5-1* seedlings grown on ½ MS medium plates containing 1% sucrose under LD

conditions were exposed to continuous light for 1 day before being transferred to liquid ½ MS-C medium supplemented with 1 µM ConA and incubated in the dark for 6 h, followed by confocal observation of the root elongation zones. Seedlings incubated under light without ConA served as a control. About 10 seedlings were observed with similar results. Scale bar, 50 µm. **f** Quantification of autophagosomes per frame in images of root tips from different genotypes shown in (**e**). Values are means ± SE from 10 images from individual roots. In (**c**, **f**), data are shown as means ± SEM. Circles indicate individual data points. Different letters indicate individual groups identified by pairwise multiple comparisons with a one-way ANOVA followed by a Tukey's test ($p < 0.05$). Source data are provided as a Source Data file.

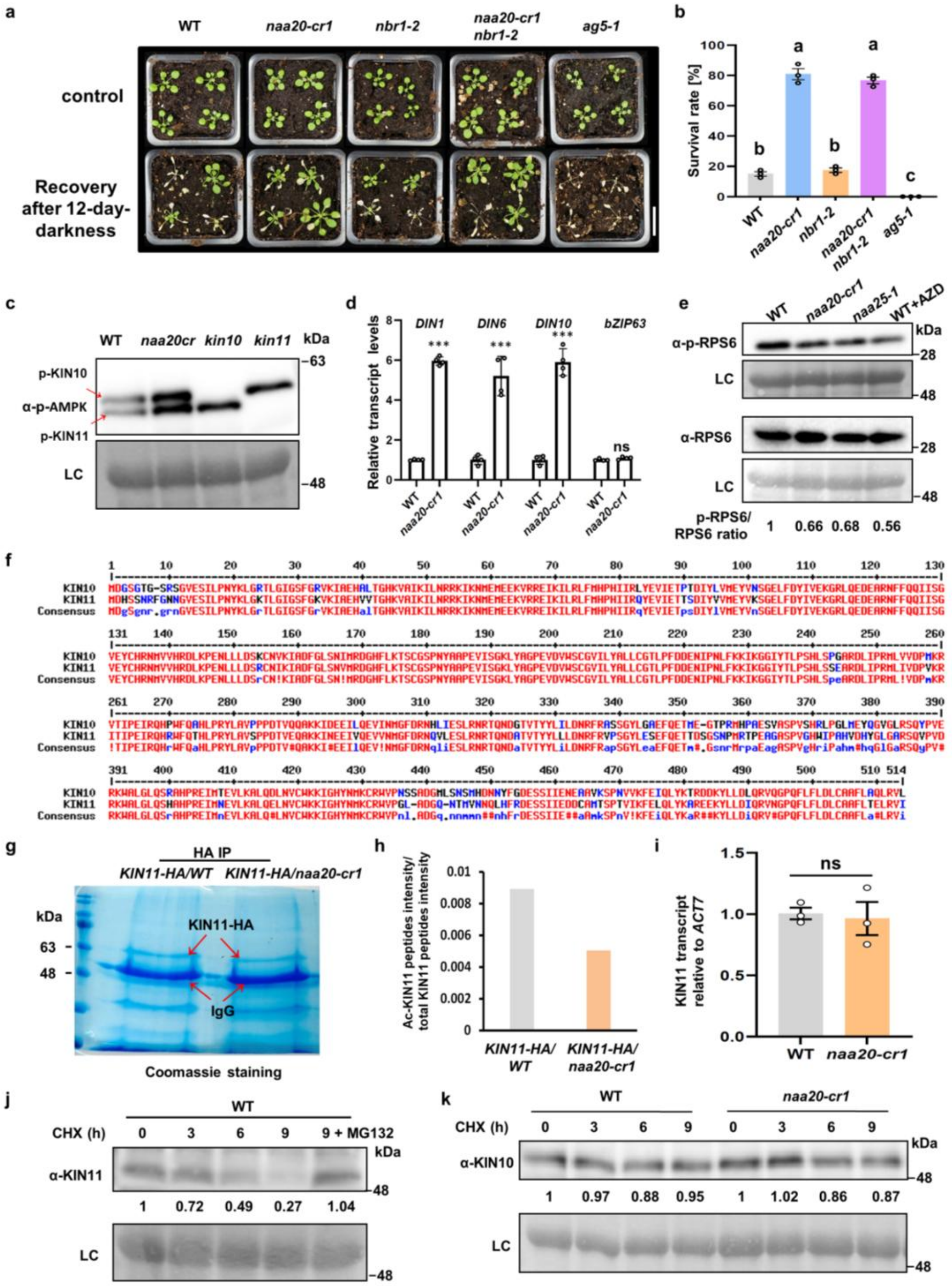

Supplementary Fig. 6 NatB-mediated protein stability of KIN11 is crucial for the autophagy activation in plants

a, **b** Representative images and survival rates of wild type, *naa20-cr1*, *nbr1-2*, *naa20-cr1 nbr1-2*, and *atg5-1* plants after 12-day-carbon-starvation followed by 12-day-recovery. Data are shown as means ± SEM (n = 3 biologically independent replicates, 40 plants for one replicate). Different letters indicate individual groups identified by pairwise multiple comparisons with a one-way ANOVA followed by a Tukey's test ($p < 0.05$). Scale bar, 2 cm. **c** Immunoblot detection of p-KIN10 and p-KIN11 in wild type, *naa20-cr1*, *kin10*, and *kin11* plants. The absent signal in *kin10* or *kin11* mutants demonstrates the specificity of the antibodies. **d** The transcript levels of *DINs* and *bZIP63* genes in wild type and *naa20-cr1*. **e** Immunoblot detection of p-RPS6 and RPS6 in wild type, *naa20-cr1*, and *naa25-1* seedlings

incubated for 90 min with DMSO or 2 μM AZD. **f** Alignment of KIN10 and KIN11 using the online MulAtlin tool (http://multalin.toulouse.inra.fr/multalin/multalin.html). Consensus is indicated in red (high), blue (low) or neutral (black). **g** Coomassie staining of HA-immunoprecipitated *KIN11-HA* (upper arrows) in wild type and *naa20-cr1* backgrounds. The lower arrows indicate IgG heavy chain. **h** Quantitation of the N-terminally acetylated KIN11 peptide (MDHSSNR) abundance in two gel bands shown in **g**, normalized to the total KIN11 peptide abundance in each sample. **i** The transcript levels of *KIN11* in wild type and *naa20-cr1*. **j** Degradation rate of KIN11 in leaves of the wild type after applying CHX or MG132. **k** Degradation rate of KIN10 in wild type and *naa20-cr1* mutant revealed by CHX chase experiments. In (**c**, **e**, **j**, **k**), amido black staining of proteins transferred to the PVDF membrane served as a loading control (LC). These experiments were repeated three times with consistent results. In (**j**, **k**), the protein levels were normalized to the control (0 h sample, set to 1). In (**d**, **i**), data represent the means ± SEM (n = 4 and 3 biologically independent samples for **d** and **i,** respectively). *Actin7* (*At5g09810*) served as the reference genes. Significance was determined by a two-sided Student's t-test ($p < 0.001$). ns, no significance. Source data are provided as a Source Data file.

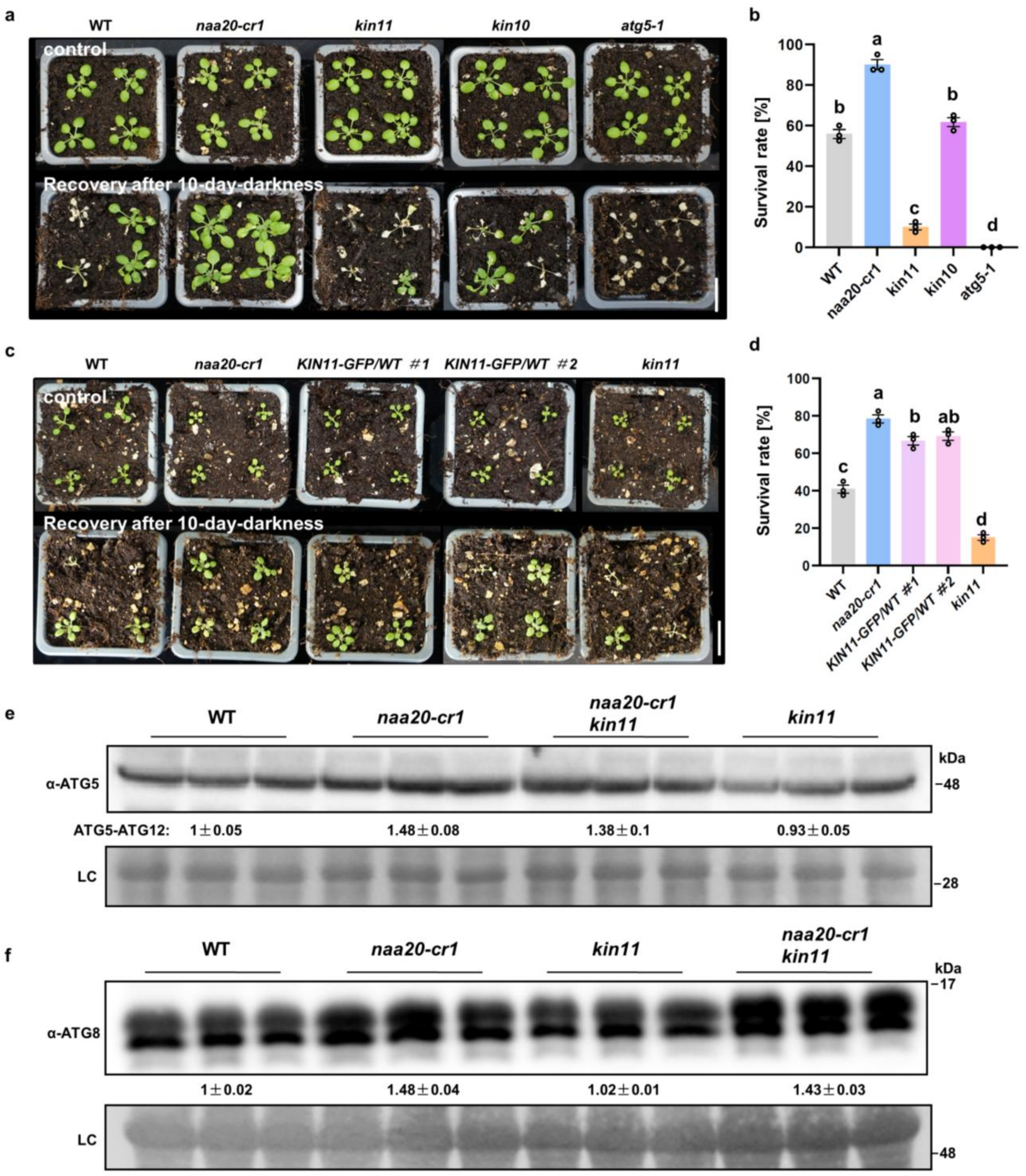

Supplementary Fig. 7 Darkness-resistance and ATG protein levels in different mutants affected in SnRK1, ATG5, and NatB

a, **b** Representative images and survival rates of wild type (WT), *naa20-cr1*, *kin11*, *kin10*, and *atg5-1* plants after 10-day carbon starvation followed by 14-day recovery. The survival rate of 40 plants grown in 10 pots was defined as one biological replicate (*n* = 3 biologically independent replicates, 120 plants in total). Scale bar, 2 cm. **c**, **d** Representative images and survival rates of wild type (WT), *naa20-cr1*, *kin11* and two independent *KIN11-GFP* overexpressing lines after 10-day carbon starvation and recovery. The survival rate of 40 plants grown in 10 pots was defined as one biological replicate (*n* = 3 biologically independent replicates, 120 plants in total). Scale bar, 2 cm. **e**, **f** Immunoblot detection of

ATG5 (**e**) and ATG8 (**f**) in wild type (WT), *naa20-cr1*, *kin11*, and *naa20-cr1 kin11* plants. Values represent the means ± SEM (*n* = 3 biologically independent samples). The protein levels were normalized to the mean WT value (set to 1). Amido black staining of proteins transferred to the PVDF membrane served as a loading control (LC). All experiments were repeated three times with consistent results. Data are shown as means ± SEM in **b, d**. Circles indicate individual data points. Different letters indicate individual groups identified by pairwise multiple comparisons with a one-way ANOVA followed by a Tukey's test ($p < 0.05$). Scale bar, 2 cm. Source data are provided as a Source Data file.
