# Supplementary material for: The ribosome-associated N-terminal acetyltransferase B coordinates global proteostasis and autophagy in plants by creating Ac/N-degrons
Source: Nat Commun. 2026 Mar 31;17:3116. doi: 10.1038/s41467-026-71208-2 (PMC13039445; doi:10.1038/s41467-026-71208-2)
Supplement: Supplementary file 2 — Description of Additional Supplementary Files [file 41467_2026_71208_MOESM2_ESM.pdf]

Description of Additional Supplementary Files

File Name: Supplementary Data 1

Description: Summary of N-terminal profiling in WT, *naa20-1* and *naa20-cr1* lines.

File Name: Supplementary Data 2

Description: Result table for the N-terminome analysis, obtained from Supplementary Data 1 containing only the N-termini with an NTA level significantly decrease (at least 20%) in *naa20-1* and *naa20-cr1*.

File Name: Supplementary Data 3

Description: Quantitative proteomic analysis of WT and *naa20-cr1*.

File Name: Supplementary Data 4

Description: Ubiquitomic analysis of WT and *naa20-cr1*.

File Name: Supplementary Data 5

Description: Primers used in this study.
